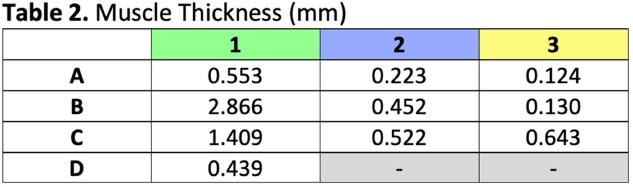# Novel Injection Technique of Botulinum Toxin to Reduce Facial Rhytids, a Cadaver Study

**DOI:** 10.1093/asjof/ojaf018.009

**Published:** 2025-05-13

**Authors:** Lamvy Le, Heidi Johng, Allen Van Beek

**Affiliations:** University of Minnesota, Minneapolis, MN; Waldorf Center for Plastic Surgery, Portland, OR; University of Minnesota, Minneapolis, MN

## Abstract

**Goals/Purpose:**

The injection of botulinum toxin is a widely performed non-invasive procedure for aesthetics of the face. Common areas to target are rhytids in the forehead, glabella, lips and crow’s feet. Using conventional methods, treatment of each area requires an average of three to five injection sites per target area. Here, we introduce and describe a novel technique for the injection of botulinum toxin to reduce the number of injection sites per treatment area, thereby reducing patient discomfort, ecchymosis and improving the patient-physician experience. To help illustrate the anatomical basis for the efficacy of this technique, we present histologic studies obtained from a fresh cadaver to further examine the anatomy between the skin and underlying facial muscles of the forehead and periorbita.

**Methods/Technique:**

All patients have the areas of maximal animation determined and marked with an eyeliner pen. Crucial to the glabellar area is the determination of the lateral extent of the corrugator supercilia and procerus muscles’ functional levels. The degree of impact of the central portion of the superior frontalis muscle on the mid and upper central forehead are marked. Care is taken not to place too much botulinum toxin laterally over the lateral frontalis, as this will result in unwanted ptosis of the lateral brow and crowd the upper lid skin resulting in a sensation of lid and forehead heaviness reported by patients. The superior and inferior portions of the lateral genu of the orbicularis oculi muscles are marked, creating a target for injection.

After marking, the plan for botulinum toxin dosage and pattern of injection is determined (Figure 1). Botulinum toxin is diluted so 0.1 ml of injected saline contains 1.2U. 4 mL of sterile saline is used for reconstitution of the 50U container and 5 mL for a 100U vial. To treat glabellar lines, 12-15 units are used with the single injection tunneling technique with three trajectories – medial and in a V-shaped pattern (Figure 1). For the forehead lines, 5-10 units are used with the single injection tunneling across the forehead (Figure 1). For the crow’s feet, 10-12 units are used for each side with the single injection tunneling technique with two trajectories (Figure 1). Because the injection volume is larger than with the multi-stick technique, the sites of injection are gently massaged to evenly distribute the injected botulinum toxin then a warm scented towel is applied over the face.

A fresh cadaver was obtained from a 51-year-old nonsmoking female with a BMI of 22.7. A full thickness specimen was obtained, including the forehead, temples, periorbita, connective tissue, muscle, and periosteum. Four sagittal sections were taken from the left face as illustrated in Figure 2 to include the central forehead, medial brow, lateral brow, and lateral orbit. These were made into paraffin blocks then processed and stained with hematoxylin and eosin (H&E) and Masson’s trichrome. Whole slides were imaged and measurements were taken using Proscia Concentriq® for Research.

**Results/Complications:**

This tunneling injection technique has resulted in decreased complaints of pain and ecchymosis, and has proven to have safe and favorable cosmetic outcomes. Patients report more rapid onset of muscle de-animation. With careful planning and targeted marking, one can create unique patterns that foster favorable or unique appearances when requested or recommended.

Our cadaver study reveals clearly defined septa connecting the skin to the underlying muscles of the forehead and periorbita (Figure 3, 4). H&E and Masson’s trichrome stains of specimens taken from the left face can be seen in Figure 3 and 4 respectively. Trichrome stains demonstrate the muscle more clearly than H&E stains, which can be seen at higher magnification (Figure 5). The H&E images better illustrate the muscle dermal connections at higher magnification (Figure 6). Skin depth to muscle and muscle thickness were measured at various points of each specimen shown in Figure 2. Skin depth to muscle and muscle thickness are recorded in Table 1 and Table 2. The thickness of the muscle appears to correlate with the dosages required for deamination at each site.

**Conclusion:**

This novel injection technique referred to as the tunneling technique was developed as an attempt to reduce the number of injection sites per treatment area thereby reducing the level of discomfort experienced with 10-20 puncture sites. This technique is operator-dependent and does require careful planning and technique to keep botulinum toxin administration in the correct plane and avoid intersecting obvious facial vessels. Our cadaver study showed many distinct septa connecting the skin to the underlying frontalis muscle, corrugator supercilii, and orbicularis oculi muscles. By injecting botulinum toxin using the tunneling technique and allowing it to diffuse in the subcutaneous plane, the product should successfully treat facial rhytids without needing to inject the muscle directly in multiple locations as done by the traditional multiple-puncture approach.

**Figure:**
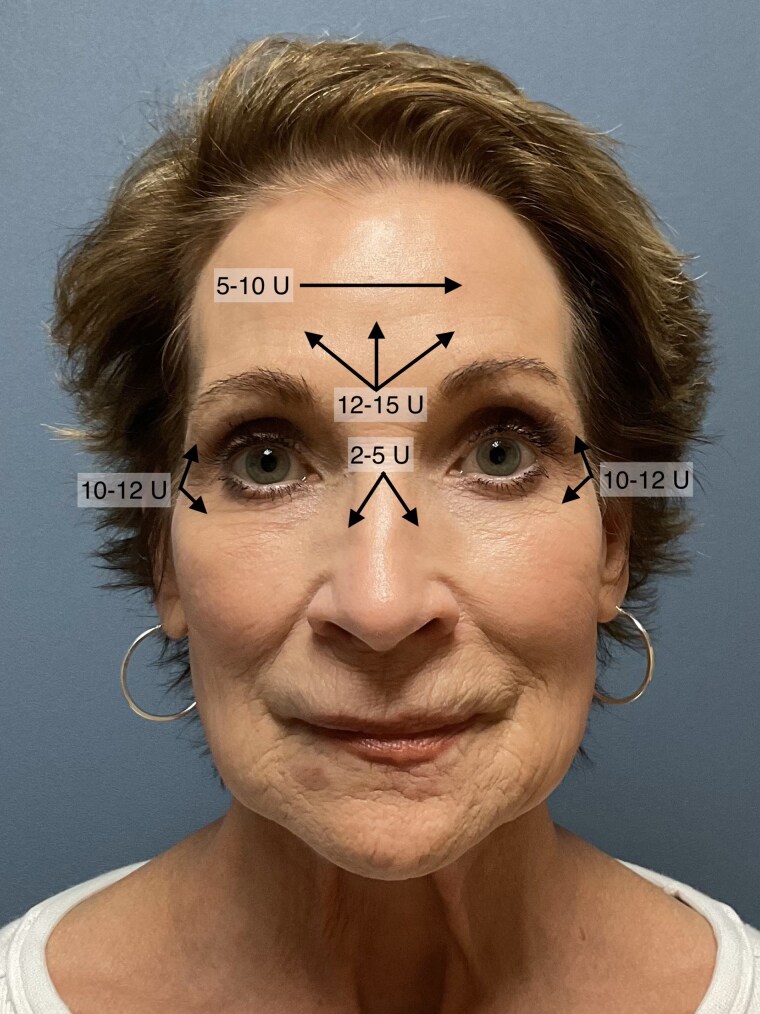

**Figure:**
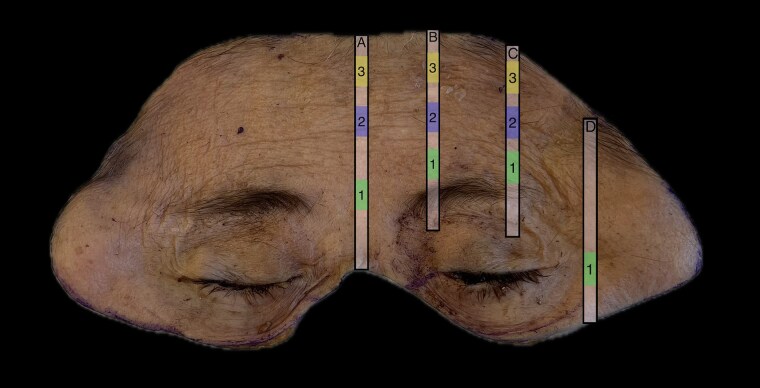

**Figure:**
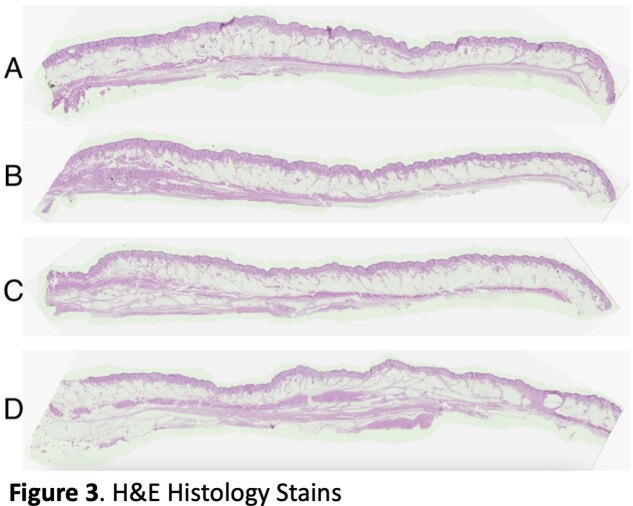

**Figure:**
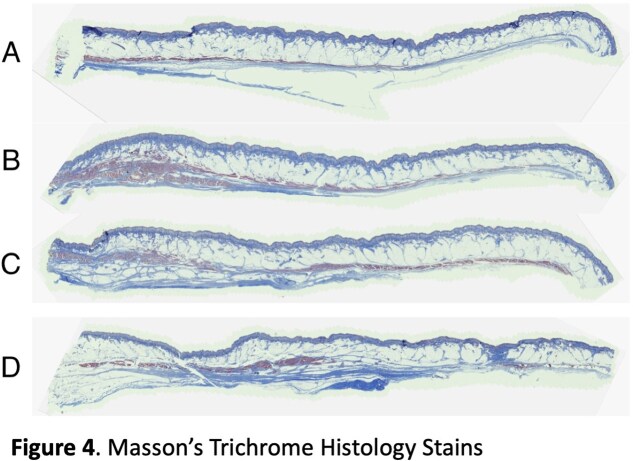

**Figure:**
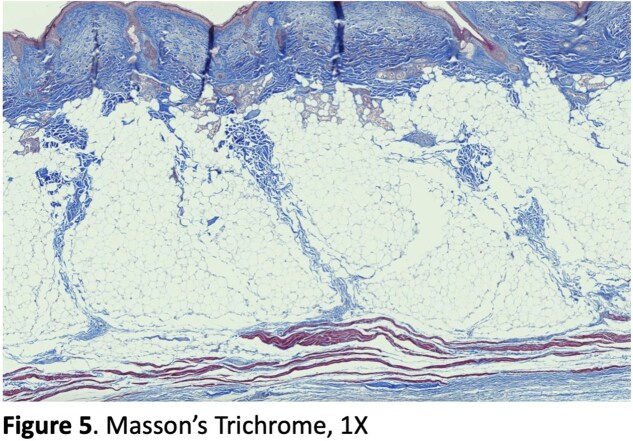

**Figure:**
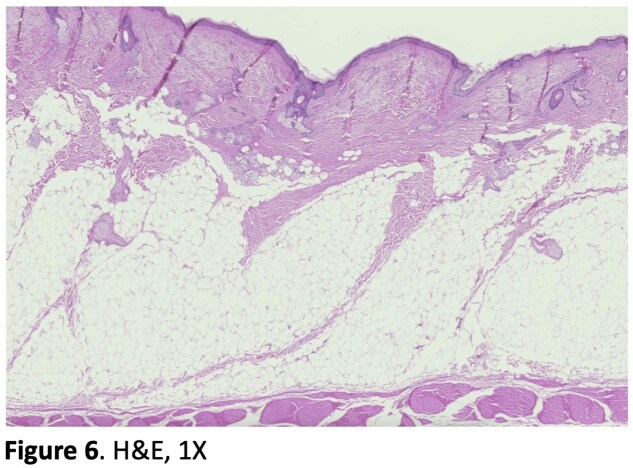

**Figure:**
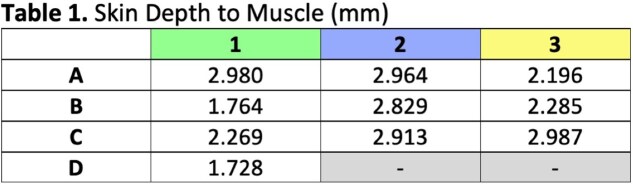

**Figure:**